# Morphological and physiological changes in *Artemisia selengensis* under drought and after rehydration recovery

**DOI:** 10.3389/fpls.2022.851942

**Published:** 2022-08-05

**Authors:** Hui-Xiong Huang, Yun Cao, Kai-Jing Xin, Rong-Hua Liang, Yi-Ting Chen, Jia-Jun Qi

**Affiliations:** ^1^School of Geography and Environment, Jiangxi Normal University, Nanchang, China; ^2^Nanchang Base of International Centre on Space Technologies for Natural and Cultural Heritage Under the Auspices of UNESCO, Nanchang, China; ^3^Key Laboratory of Poyang Lake Wetland and Watershed Research, Ministry of Education, Nanchang, China

**Keywords:** wetland plants, drought stress, *Artemisia selengensis*, pot experiment, compensation mechanism

## Abstract

Changes in global climate and precipitation patterns have exacerbated the existing uneven distribution of water, causing many plants to face the alternate situation of drought and water flooding. We studied the growth and physiological response of the wetland plant *Artemisia selengensis* to drought and rehydration. In this study, *Artemisia selengensis* seedlings were subjected to 32.89% (SD), 47.36 % (MD), 60.97% (MID), and 87.18 % (CK) field water holding capacity for 70 days, followed by 14 days of rehydration. The results showed that drought inhibited the increase of plant height, basal diameter, and biomass accumulation under SD and MD, but the root shoot ratio (R/S) increased. Drought stress also decreased the content of total chlorophyll (Chl), chlorophyll a (Chl-a), chlorophyll b (Chl-b), and carotenoid (Car). Soluble sugar (SS) and proline (Pro) were accumulated rapidly under drought, and the relative water content (RWC) of leaves was kept at a high level of 80%. After rehydration, the plant height, basal diameter, biomass, and R/S ratio could not be recovered under SD and MD, but these indicators were completely recovered under MID. The RWC, Chl, Chl-a, Chl-b, Car, and osmotic substances were partially or completely recovered. In conclusion, *Artemisia selengensis* not only can improve drought resistance by increasing the R/S ratio and osmotic substances but also adopt the compensatory mechanism during rehydration. It is predictable that *A. selengensis* may benefit from possible future aridification of wetlands and expand population distribution.

## Introduction

During the life cycle of plants, they may undergo frequent periods of water deficit, and drought is one of the most serious abiotic stresses that limit plant growth and reduce yield (Cohen et al., 2021; Yang et al., 2021). Soil water loss induced by drought not only affects the survival, growth, and distribution of plants (Choat et al., 2018; Jung et al., 2020) but also their physiological and metabolic functions (Gupta et al., 2020). Due to recognized global climate change, regions where drought could be ignored in the past will also confront the problem. The trend of aridity exacerbated by global warming (IPCC., 2021) has led to the threat of drought for many plants worldwide. To survive and reproduce, most plants have evolved a variety of adaptive mechanisms and strategies to address drought stress (Seleiman et al., 2021). For instance, plants can adapt to water deficit by inducing a variety of morphological, physiological, biochemical, and molecular changes (Toscano et al., 2019; Sun et al., 2020). Drought tolerance is defined as the ability to grow, flower, and produce at low water availability (Bandurska, 2022). However, drought and rehydration often occur consecutively in plant life history, and the ability of plants to maintain physiological functions under low water status and recover rapidly after stress removal is vital to ensure growth and development under intermittent drought events. In this context, studying the adaptation mechanisms of plants under drought and their recovery after rehydration can not only illuminate the drought tolerance mechanisms of plants but also have important implications for plant propagation and scientific utilization.

Inhibition of plant morphological growth is the most obvious feature after drought (An and Liang, 2012) and is an extrinsic expression of the plant's physiological response. Various previous studies have suggested that drought can negatively affect the plant growth rate, biomass, and yield (Lipiec et al., 2013; Gupta et al., 2020; Liu et al., 2021). Additionally, since the plant root is the main organ that uptakes water and inorganic salts from the soil (Kim et al., 2020) and the aboveground part is mainly used for photosynthesis, the biomass allocation between them is also considered to be an important way of plant adaptation to the environment (Eziz et al., 2017; Du et al., 2020). Plants respond to water deficiency by increasing the flow of carbon dioxide assimilates to the root system, thereby increasing the proportion of root biomass (Chen et al., 2022). Such reallocation of photo assimilates is regarded as an effective adaptation mechanism that minimizes the evaporative area of the plant canopy (Díaz-López et al., 2012), while improving the rate of water uptake from dry soil by enlarging the exposure area (Mo et al., 2015). Because of water deficit, leaves suffer permanent or temporary changes in morphology and anatomy. Among them, relative water content (RWC) is the relatively obvious physiological change of plant leaves, which is the ratio of actual water content to saturated water content of plant tissues (Kumar and Sharma, 2010). Previous studies have shown that drought often leads to decreased RWC in plant organs (Abid et al., 2018; Amnan et al., 2022). Besides the limitation of water availability, drought results in a reduction of plant carbon assimilation, which greatly depends on photosynthesis (Ding et al., 2018), which is one of the most sensitive life processes of plants in response to drought. It is well-known that photosynthetic pigments, especially chlorophyll, play a key role in plant photosynthesis. The decrease in chlorophyll (Chl) content is a commonly observed phenomenon under drought (Chen et al., 2016; Miao et al., 2020), but the Chl content in some plants tends to increase (Pirzad et al., 2011). Carotenoids (Car) are auxiliary pigments essential for photosynthesis, and their response to drought is somewhat different from that of Chl. Many studies have shown that drought increases Car content (Sedghi et al., 2012; Saeidnia et al., 2018), and plants of drought-tolerant genotypes maintain higher Car content than drought-sensitive genotypes under drought (Shah et al., 2011). It has also been reported that drought significantly reduces Car content (Mibei et al., 2017; Khodabin et al., 2020).

Osmotic adjustment, as an important physiological mechanism for plant adaptation to drought stress, has become one of the most active fields of drought resistance physiology research (Liu et al., 2011; Shemi et al., 2021). Plants under drought maintain appropriate levels of stomatal opening for photosynthesis through osmotic substances. These osmotic substances include organic solutes synthesized in the cell, mainly proline (Pro), soluble protein (SP), and soluble sugar (SS). SS (sucrose, glucose, and fructose) plays an important role in maintaining the structure and growth of plants. Previous studies have shown that SS acts as osmoprotectants, regulating osmotic pressure, providing membrane protection, scavenging toxic reactive oxygen species, and safeguarding the stability of plant enzymes/proteins (Sami et al., 2016). Pro accumulation is a common physiological response in many plants in response to biotic and abiotic stresses; it regulates cellular osmotic pressure, stabilizes the structure of proteins and cell membranes, is a protective agent for enzymes, and is a free radical scavenger and antioxidant (Kishor and Sreenivasulu, 2014). Besides being an osmotic substance, SP is also the key nutrient. Their increase and accumulation improve the water-holding capacity of cells and protects intracellular life substances and biofilms. Previous studies have shown that drought led to a gradual decrease in water content in barley and an increase in SS and Pro content (Bandurska et al., 2017; Chang et al., 2021). Studies on Hemerocallis showed that SP content increased significantly under moderate drought, and SS content decreased under severe and moderate drought (Chen Y. H. et al., 2021). Indigenous proline is the most drought-sensitive osmotic substance in fennel, and external application of Pro also improves drought resistance (Zali and Ehsanzadeh, 2018). Therefore, SP, Pro, and SS were used as important indicators for the selection of drought-resistant varieties (Wang Q. et al., 2019).

In general, plant responses to drought and rehydration vary with stress duration, intensity, plant growth stage, and rehydration rate (Giorio et al., 2018; Hao et al., 2019; Georgieva et al., 2020). Recently, studies on the effects of drought and rehydration on plants have focused on various agricultural and cash crops, e.g., wheat, rice, soybean, and medicinal herbs (Wu et al., 2016; Dong et al., 2019; Voronin et al., 2019; Auler et al., 2021). Arid and mesophytic plants are the major subjects of study in this field, but there are limited studies related to Wetland plants. Considering that a warmer climate will increase the frequency, duration, and intensity of drought, some wetlands are facing degradation problems, such as area reduction, functional deterioration, and biodiversity decrease (Song et al., 2011; Hu et al., 2019; Jeelani et al., 2020), and wetland plants may be impacted by intermittent drought and rehydration. Therefore, it is important to assess the effects of drought on wetland plant functions and the mechanisms of plant responses after rehydration. The related knowledge is essential for modeling and predicting the fate of wetland ecosystems under future climate conditions.

*Artemisia selengensis* (*A. selengensis*) is a perennial wet plant of the genus *Artemisia* in the family *Asteraceae*, widely distributed in wetlands, marshes, wet meadows, and freshwater lake meadows of the middle and lower reaches of the Yangtze River in China (Zhang et al., 2018). *A. selengensis* has been used as food and herbal medicine in China for a long time. The tender stems of *A. selengensis* are edible and nutritious. Its main active components, such as polyphenols, flavonoids, volatile oils, polysaccharides, and terpenoids, have anti-inflammatory, antibacterial, and antioxidant effects (Zhang M. et al., 2015; Wang et al., 2020). *A. selengensis* is a typical dominant species in Poyang Lake, the largest freshwater lake in China (Wang et al., 2017), and its growth and distribution are affected by water conditions (Fan et al., 2019). However, due to the impact of climate change and human activities (Ye et al., 2018; Liu et al., 2020), the spatial and temporal distribution of water in Poyang Lake has become more uneven, and extreme droughts occur frequently (Zhang Z. X. et al., 2015; Li Y. L. et al., 2021). As a result, *A. selengensis* may need to experience a long drought and a wet season of rehydration in its life cycle. To the best of our knowledge, however, little information on mechanisms of drought tolerance and post-drought rehydration in *A. selengensis* is available. Therefore, we conducted a drought and rehydration experiment to explore the morphological and physiological responses of *A. selengensis* to different drought levels and rehydration. We assumed that (1) long-term drought would inhibit the normal growth of *A. selengensis* and lead to changes in plant RWC, photosynthetic pigments and osmotic substances, (2) *A. selengensis* might be a drought-tolerant plant, and its morphophysiological parameters could all be completely or partially recovered after rehydration.

## Materials and methods

### Experimental materials

In this study, *A. selengensis* seedlings were used as experimental materials. The wild *A. selengensis* with relatively uniform growth and strong stalks was collected from Poyang Lake National Wetland Park, Jiangxi Province, China (29°05′30″N, 115°55′39″E) on January 13, 2021. *A. selengensis* seedlings were cut into sections about 5-cm long, with one or two side buds per section. The stems were planted in plastic pots (35 cm × 26 cm × 13 cm), with the lower part of the stems buried in the soil about 2 cm, and 10 plants were planted in each pot for pre-culture. Before the drought experiments began, all the plants were well-watered every day. The substrate soil used for preculture was swamp soil extracted from Aixi Lake National Wetland Park in Nanchang City, China, where *A. selengensis* grows. After removing impurities such as dead branches and leaves, the soil is mixed together for use. To calculate the field water-holding capacity of the soil, the soil samples were soaked in water for 24 h until saturated and weighed. Afterward, the soil was dried to a constant weight in an oven at 105°C. The soil water-holding capacity was calculated:


$$\begin{array}{l} FC\left(\%\right)=\frac{\left(SW-DW\right)}{DW}\times 100 \end{array}$$

where FC is field water-holding capacity of soil, SW is weight of saturated soil, and DW is weight of dried soil.

The basic properties of the soil were: organic matter content of 39 g kg^−1^, the total nitrogen content of 1.8 g kg^−1^, pH of 5.4, and field water-holding capacity (FC) of 43.8%.

### Experimental design

**Figure 1:**
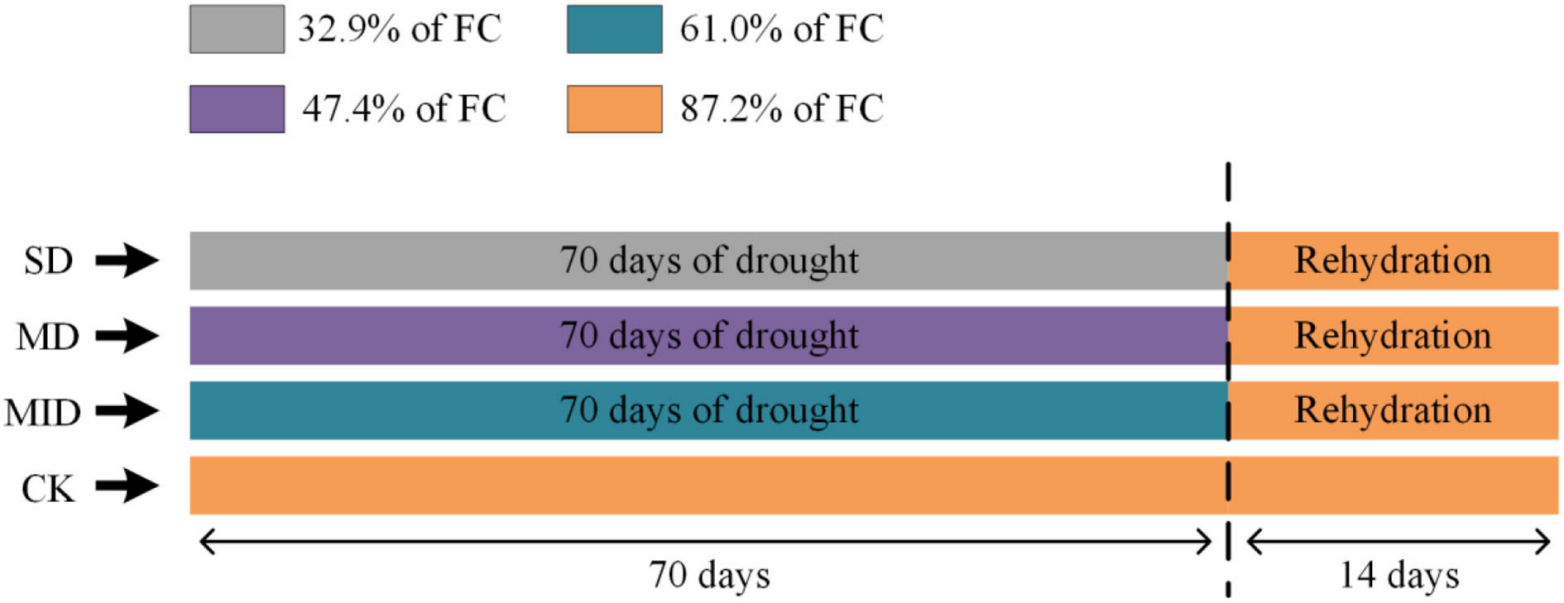
The water treatment design was carried out using *A. selengensis* as the testing material. FC, field water-holding capacity; SD, severe drought group; MD, severe drought group; MID, mild drought group; CK, control group. After the drought experiment (70 days), SD, MD, and MID treatments were re-watered to the control level (14 days). Samples were taken every 14 days for measurement.

The experiment was conducted in the plant sunlight culture room at the Key Laboratory of Poyang Lake Wetland and Watershed Research, Ministry of Education, Jiangxi Province, China. The experiment started on March 23, 2021; the soil water content of pots was adjusted to the set drought level by controlling the watering volume and natural evaporation 1 week before the experiment. In the drought experiment, four relative soil water contents were set up, and each treatment was conducted with three replicates (each water gradient has three pots of plants), totaling 12 pots of *A. selengensis* seedlings. The details of relative soil water content are as follows: soil of the control group (CK) contained 87.2% of maximum field water-holding capacity (FC); soil of the mild drought group (MID) contained 61% of FC; soil of the moderate drought group (MD) contained 47.4% of FC; soil of the severe drought group (SD) contained 32.9% of FC (Figure 1). Soil relative water content (%) = (soil water content/field water-holding capacity) × 100, where soil water content is measured using an HH2 soil moisture meter (Delta-T, UK). When measuring soil moisture, insert the moisture meter probe into the potting soil about 10 cm. In principle, sensors measured the dielectric constant of soil and then converted these data to the values of water content.

To maintain the soil moisture at the setting level of each treatment group, the soil water content in pots was measured daily at 17:00 by a soil moisture meter, and then water was supplemented quantitatively. The drought experiment finished on 1 June, totaling 70 days, and then the plants were watered to make the drought group's soil water content up to 85-90% of FC (CK). The rehydration experiment was conducted for 14 days, and the treatment groups were watered daily at 17:00 to maintain the soil moisture at the set value. The experiment was duration of 84 days in total (Figure 1). During the experiment, the culture room was lighted naturally, the average air temperature was 26.1 ± 5.9°C, and the average air humidity was 77.09 ± 14.08%.

### Indicators measurement

#### Morphological indicators

##### Measurements of plant height and basal diameter

Plant height and basal diameter of *A. selengensis* were measured on days 0, 14, 28, 42, 56, 70 of drought treatment, and Day 14 after rehydration (RH14). Plant height was measured with tape measure, and the basal diameter was measured with Vernier calipers. The plant height and basal diameter measurements of each moisture treatment were conducted with three replicates.

##### Measurements of biomass and root shoot ratio

**Figure 2:**
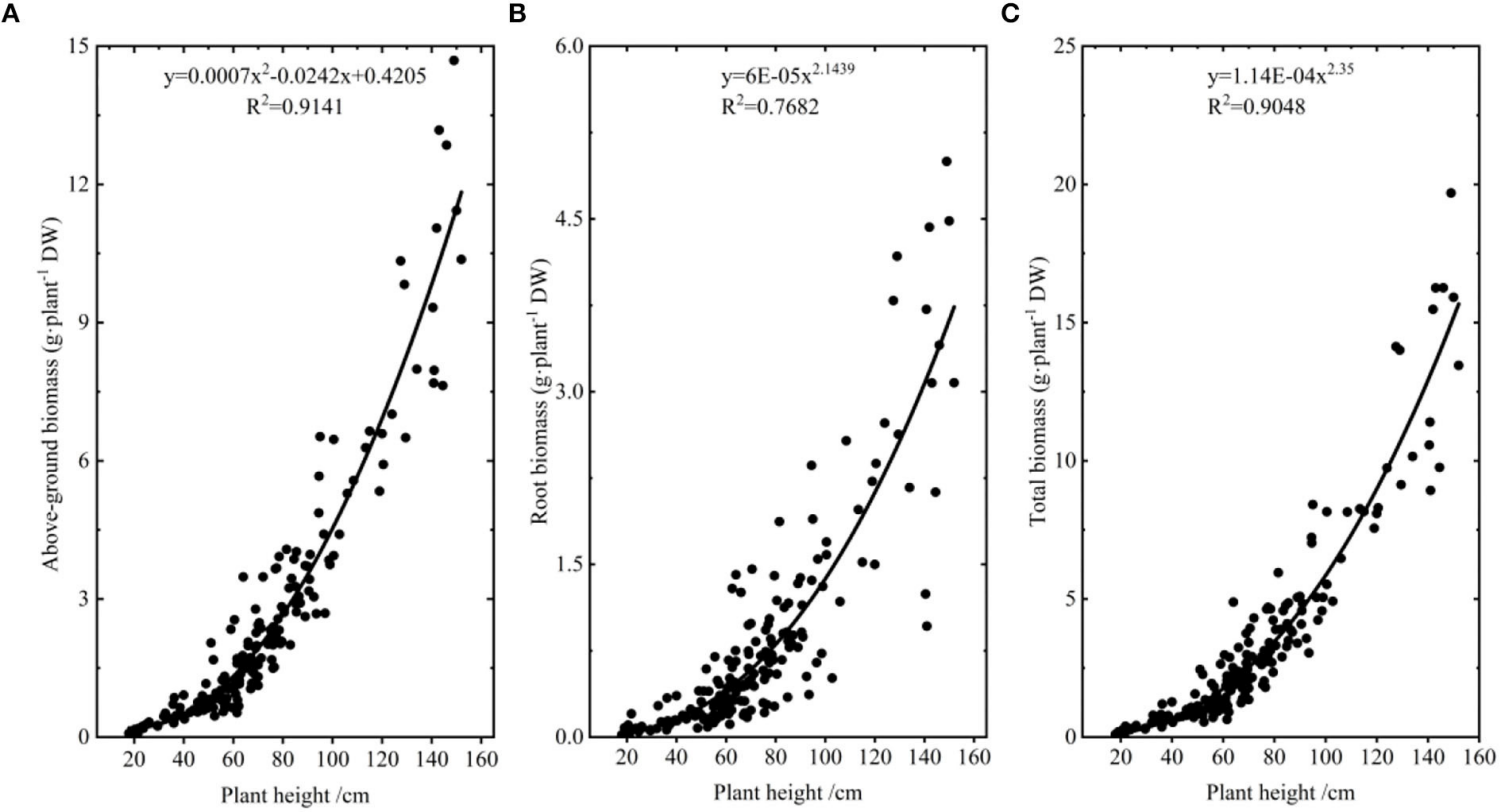
The biomass model of *A. selengensis*. **(A)** The aboveground biomass model. **(B)** The root biomass model. **(C)** The total biomass model.

In order not to disturb *A. selengensis* excessively, biomass was calculated by the biomass modeling method (Luo et al., 2017). We collected *A. selengensis* in the field at the early, middle, and late stages of its growth, and brought it back to the laboratory (wild *A. selengensis* was only used to model biomass). The soil water content of the sampling site was determined to be 38 ± 10.6% using the soil moisture meter, with a range of 23.1–54.3%. We cut each plant above and below-ground parts, cleaned them of dust and dirt, measured the plant height and basal diameter, and then bagged and numbered them. Afterward, these samples were put in oven at 75°C for 72 h to dry weight (DW), and the aboveground and root biomass of each plant was obtained. In regression analysis of plant height, basal diameter, and biomass, we found a high correlation between plant height and biomass, so we chose plant height to construct a biomass model, and the fitting curves of aboveground biomass, root biomass, total biomass, and plant height are shown in Figure 2.

After calculating the aboveground and root biomass from the biomass model, the root shoot ratio (R/S) was calculated by the following formula.


$$\begin{array}{l} R/S=\frac{B_{b}}{B_{a}} \end{array}$$

where R/S is the root shoot ratio, B_b_ is root biomass (g/plant DW), and B_a_ is aboveground biomass (g/plant DW).

#### Measurement of physiological indicators

##### Sampling

There were seven sampling times, including 0, 14, 28, 42, 56, 70 days after drought treatment, and 14 days after rehydration treatment (RH14). And the sampling site was the middle leaf of the plant. We collected leaves from the three pots of the plants, and then the three samples of the leaves were collected to obtain average values.

##### Measurement of photosynthetic pigment

The photosynthetic pigment content of plant leaves was measured by 95% ethanol extraction. Grind, 0.2–0.5-g fresh leaves with 95% ethanol and filter the extract into a 25-ml brown volumetric flask to volume. Three repetitions of each treatment were measured. With 95% alcohol as the control group, the absorbance was measured by spectrophotometer at wavelengths of 665, 649, and 470 nm, respectively (Li M. et al., 2021). The formulas for calculating photosynthetic pigment content based on absorbance are as follows.


Ca=13.95A665-6.88A649Cb=24.96A649-7.32A665                        CT=Cb+Cb  Cx=1000A470-2.05Ca-114.8Cb245                           CP=C × VMf

where C_a_, C_b_, C_T_, and C_x_ are the concentrations of chlorophyll a, chlorophyll b, total chlorophyll, and carotenoids, respectively (mg/L); A_665_, A_649_, and A_470_ are the absorbance of sample extracts at 665, 649, and 470 nm, respectively; C_P_ is the content of each pigment per unit dry weight (mg/gDW), C is the pigment in sample extracts concentration (mg/L), V is the volume of the sample extract (L), Mf is the dry weight of the leaf sample (g), and the dry weight was calculated from the data of leaf water content.

##### Measurement of relative water content of leaves

After sampling, the fresh weight (FW) of the leaves was measured immediately. Then, weighed leaves were soaked in distilled water in darkness for 24 h, and saturation weight (SW) was measured. Finally, the samples were dried in an oven at 85°C for a duration of 48 h to constant weight, and then dry weight (DW) was measured. Three repetitions of each treatment were measured. The relative water content was calculated by the following formula (Rasool et al., 2020).


$$\begin{array}{l} RWC\left(\%\right)=\frac{FW-DW}{SW-DW}\times 100 \end{array}$$

where RWC is the relative water content (%), FW is fresh weight of the leaf (g), SW is the dry weight (g), and TW is saturation weight (g).

##### Measurement of osmolytic substances

Soluble protein content was determined by the Coomassie brilliant blue G-250 method (Bradford, 1976). Approximately, 0.2 g of fresh leaves was grinded into a homogenate with 6 ml of phosphoric acid buffer solution (pH 7.8). The samples were mixed and put at room temperature for 1 h. And the mixture was centrifuged at 4,000 r/min for 20 min. Transferring upper supernatant to a 10-ml volumetric flask, dilute with distilled water to volume, and obtain the solution to be assayed. Approximately, 0.1 ml of protein extract and 5 ml of Coomassie brilliant blue G-250 protein solution were drawn into the test tubes, mixed thoroughly and left for 2 min, and the absorbance was obtained by colorimetry at 595 nm. Three repetitions of each treatment were measured. And the protein content of samples was calculated by the following formula.


$$\begin{array}{l} C_{S}=\frac{X*V_{T}*N}{W*V_{S}*1000} \end{array}$$

where C_S_ is the soluble protein content (mg/gDW); X is the standard curve value (ug) according to the absorbance; V_T_ is the total volume of the extract (ml); W is the dry weight of the sample (g), and the dry weight was calculated from the data of leaf water content; V_S_ is the volume of solution to be measured (ml); N is the dilution multiple.

The soluble sugar was determined by the anthrone colorimetric method according to the *Experimental Principles and Techniques of Plant Physiology and Biochemistry* (Wang, 2006). About 0.2 g of fresh plant leaves was weighed, cut, and put into test tubes; 5 ml of distilled water was added, sealed, and bathed in boiling water for 30 min; and the extract was filtered into a 25-ml volumetric flask and distilled water was added to volume.

In turn, 0.5 ml of sample extract, 1.5 ml of distilled water, 0.5 ml of anthrone ethyl acetate solution, and 5 ml of concentrated sulfuric acid were drawn into a test tube, shaken thoroughly and then held in a boiling water bath for 1 min. Then remove and cool to room temperature, and measure its absorbance at 630 nm. The sugar content in 0.5 ml of the extract was obtained from the standard curve of sucrose solution, and the soluble sugar content of the sample was calculated according to the following formula.


$$\begin{array}{l} C_{S}=\frac{C\times V_{T}\times N}{W\times V_{S}\times 10^{3}} \end{array}$$

where C_S_ is the soluble sugar content (mg/gDW); C is the amount of sugar (ug) found from the standard curve; V_T_ is the total volume of sample extract (ml); V_S_ is the volume of extraction solution taken for the assay (ml); N is the dilution multiple; W is the dry weight of the sample (g), and the dry weight was calculated from the data of leaf water content.

The proline content was measured by the acidic ninhydrin method (Bates et al., 1973). Approximately, a 0.2-g fresh leaf sample was placed in a test tube, and 5 ml of 3% sulfonic acid solution was added, sealed and boiled in a water bath for 10 min, and cooled to room temperature. Then, 2 ml of ninhydrin and 2 ml of glacial acetic acid were added to 2 ml of supernatant; the test tubes containing the reaction mixture were kept in a boiling water bath for 30 min and cooled. Then, 4 ml of toluene was added to the tube and shaken vigorously for 30 s; it was left for a few moments and then centrifuged. The upper solution was drawn in a cuvette, and the absorbance was measured at 520 nm using spectrophotometer. The concentration of proline in 2 ml of the extract was found from the proline standard curve, and the proline content in the sample was calculated as follows.


$$\begin{array}{l} C_{P}=\frac{X*V_{T}}{W*V_{S}} \end{array}$$

where C_P_ is proline content (ug/gDW); X is proline content (ug/2 ml) in 2 ml of assay solution found from a standard curve; V_T_ is total volume of extract (ml); V_S_ is volume of extraction solution taken for assay (ml); W is dry weight of a sample (g), and the dry weight was calculated from the data of leaf water content.

### Data statistics

The experimental data were analyzed using Excel 2019 and SPSS 24.0 (SPSS Inc., Chicago, IL, USA) software, and expressed as mean ± standard error (SE). Two-way ANOVA was applied to detect the main effects and interactions of soil water treatment and test duration on the functional traits of plants. One-way ANOVA was used to analyze the differences in growth and physiological indicators among different treatments or duration., and the Duncan's multiple comparison test was used to make multiple comparisons at the *p* < 0.05 level. The significance of differences among groups was expressed with different letters. All figures in this paper were created using Origin 2021b (Originlab Co., Northampton, MA, United States).

## Results

There were significant differences in the measured indicators between different soil moisture treatments and test durations employed in this study (Table 1). Effects of soil moisture treatments and treatment durations were observed for plant height, basal diameter, biomass, R/S ratio, RWC, chlorophyll a (Chl-a), chlorophyll b (Chl-b), chlorophyll (Chl), SS, and, which were significant (*p* < 0.01). In addition, the interaction effect of the two factors had significant effects on plant height, biomass, R/S, RWC, photosynthetic pigment, and osmotic substances (*p* < 0.01). Then, we compared the differences in indicators of *A. selengensis* in different water treatments and durations.

**Table 1:** F-values from two-way ANOVA for soil water treatment (T) and treatment duration (D) among morphological and physiological indexes of *A. selengensis*.

**Test indicators**	**T**	**D**	**T × D**
Plant height	65.36**	107.30**	5.37**
Basal diameter	7.24**	25.13**	0.73^ns^
Biomass	51.97**	64.18**	6.62**
R/S	63.96**	180.44**	3.01**
RWC	39.31**	32.13**	3.23**
Chl-a	189.77**	280.86**	76.25**
Chl-b	378.65**	927.90**	149.03**
Chl	192.64**	309.73**	77.16**
Car	73.72**	111.46**	13.18**
SP	32.59**	446.16**	23.16**
SS	175.48**	885.48**	60.64**
Pro	705.81**	979.77**	470.85**

WC, relative water content, %; Chl-a, chlorophyll a, mg/g DW; Chl-b, chlorophyll b, mg/g DW; Chl, chlorophyll, mg/g DW; Car, carotenoid, mg/g DW; SP, soluble protein, mg/g DW; SS, soluble sugar, mg/g DW; Pro, proline, ug/g DW. NS, not significant at α = 0.05;

* significant at α = 0.05;

** significant at α = 0.01.

### Effects of drought and rehydration on the growth of *A. selengensis*

#### Effects of different drought and rehydration on plant height of *A. selengensis*

**Figure 3:**
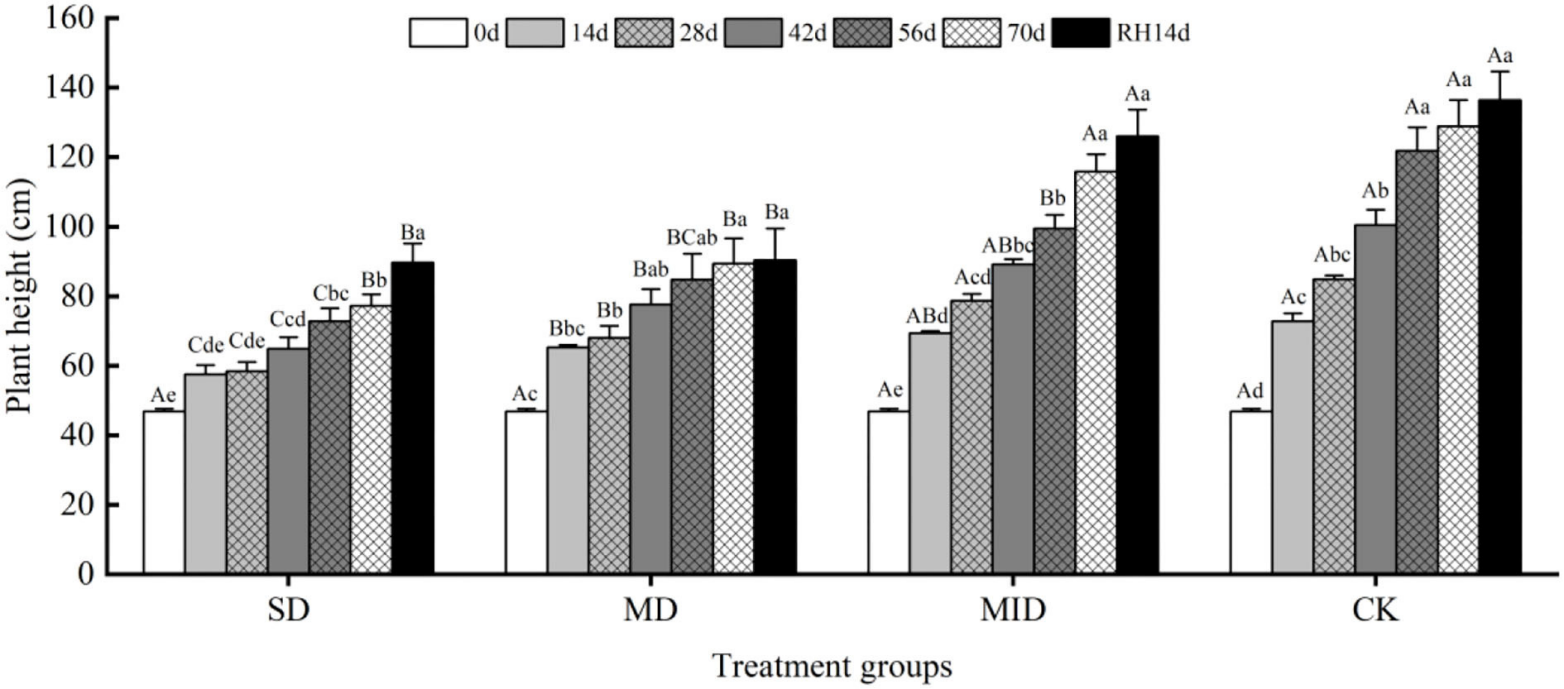
Plant height during the drought and rehydration for *A. selengensis*. 0 day, 14 days, 28 days, 42 days, 56 days, 70 days, and RH, 14 days, respectively represent 0th, 14th, 28th, 42nd, 56th, 70th under drought and 14th day of rehydration. Severe drought group (SD), moderate drought group (MD), mild drought group (MID), control group (CK). Different capital letters stand for significant differences between different drought groups (*p* < 0.05), and different lowercase letters stand for significant differences between different experimental times (*p* < 0.05).

The effects of various levels of drought and then rehydration on plant height of *A. selengensis* are shown in Figure 3. Overall, the growth trend of *A. selengensis* under different drought levels was consistent and all increased with time. The groups showed different degrees of growth slowdown when soil moisture was deficient. The *A. selengensis* of SD and MD grew rather slowly, and their plant height was significantly different (*p* < 0.05) from MID and CK. Days 0–14 were the period of the fastest plant height increase. The plant height of MD, MID, and CK increased by 18.50, 22.57, and 25.97 cm in 14 days, respectively, but only 10.67 cm for severe drought. At this time, the plant height of SD and MD was significantly lower than CK (*p* < 0.05). There was a significant difference in plant height between SD and MD on Days 14–42 (*p* < 0.05), but the significant difference was eliminated at Days 56–70 (*p* > 0.05), indicating that, with the extension of drought time, the increase of plant height in the MD was inhibited. On Day 70 of drought, plant height of SD increased by 30.43 cm compared to initial value, while plant height in MD, MID, and CK increased by 42.57, 68.93, and 82.00 cm, respectively. At this time, there was no significant difference in plant height between SD and MD (*p* > 0.05), and there was a very significant difference in plant height between MID and CK (*p* < 0.01). Severe and moderate drought inhibited the increase of *A. selengensis* height.

After rehydration, the plant height of *A. selengensis* kept growing and finally reached the maximum on Day 14. On Day 14 of rehydration (end of the experiment), there was a significant difference (*p* < 0.01) between the plant heights of SD, MD with MID, CK. It indicates that the plant height of *A. selengensis* could recover after rehydration at low drought levels, while this process became more difficult at higher degrees of drought.

#### Effects of drought and rehydration on basal diameter of *A. selengensis*

**Figure 4:**
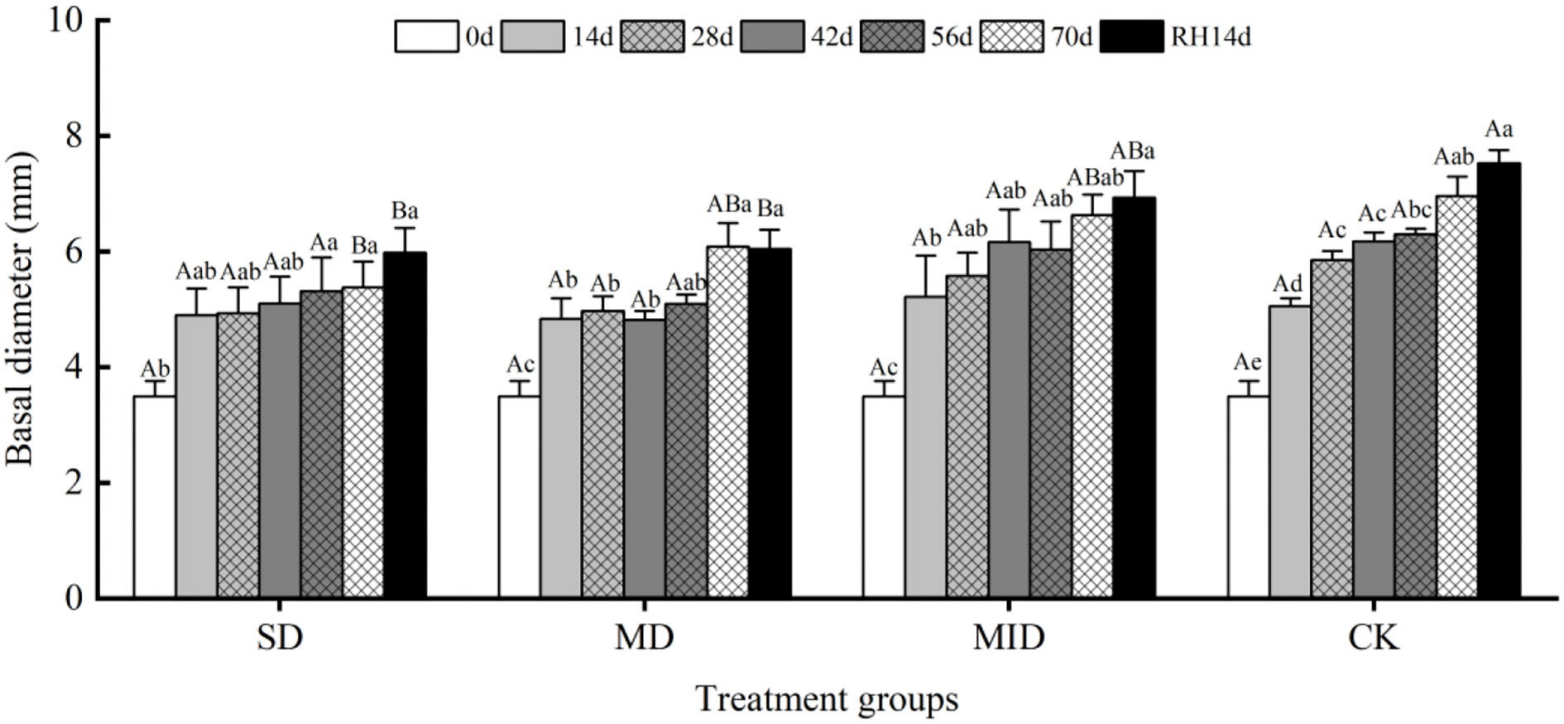
The basal diameter during the drought and rehydration for *A. selengensis*. 0 day, 14 days, 28 days, 42 days, 56 days, 70 days, and RH, 14 days respectively represent 0th, 14th, 28th, 42nd, 56th, 70th under drought and 14th day of rehydration. The severe drought group (SD), the moderate drought group (MD), the mild drought group (MID), the control group (CK). Different capital letters stand for significant differences between different drought groups (*p* < 0.05), and different lowercase letters stand for significant differences between different experimental times (*p* < 0.05).

The effects of drought and then rehydration on the basal diameter of *A. selengensis* are shown in Figure 4. Overall, the basal diameter of all groups showed an increment trend with time, while the drought groups showed a lower rate of increase than CK. Similar to plant height, the basal diameter of *A. selengensis* increased rapidly on Days 0–14, SD, MD, MID, and CK increased by 1.40, 1.34, 1.72, and 1.56 mm, respectively. There was no significant difference in the basal diameter between groups on Days 0–56 (*p* > 0.05). On Day 70, the basal diameters under SD, MD, MID, and CK were 5.38, 6.08, 6.63, and 6.96 mm, respectively. The basal diameter of the SD group was significantly lower than that of MD, MID, CK (*p* < 0.05); there was no significant difference between MD, MID, and CK (*p* > 0.05). It can be seen that water deficit of plants due to severe drought seriously affected the growth of the stems.

After rehydration, the basal diameter of different groups showed an overall increasing trend, but the change was not significant compared with that before rehydration (*p* > 0.05). After 14 days of rehydration, the basal diameters of SD and MD were significantly different from CK (*p* < 0.05), while the MID was not significantly different from CK (*p* > 0.05). It indicates that severe and moderate drought had a serious negative effect on the stems of *A. selengensis*, and rehydration did not recover them to control levels.

#### Effects of drought and rehydration on biomass of *A. selengensis*

**Figure 5:**
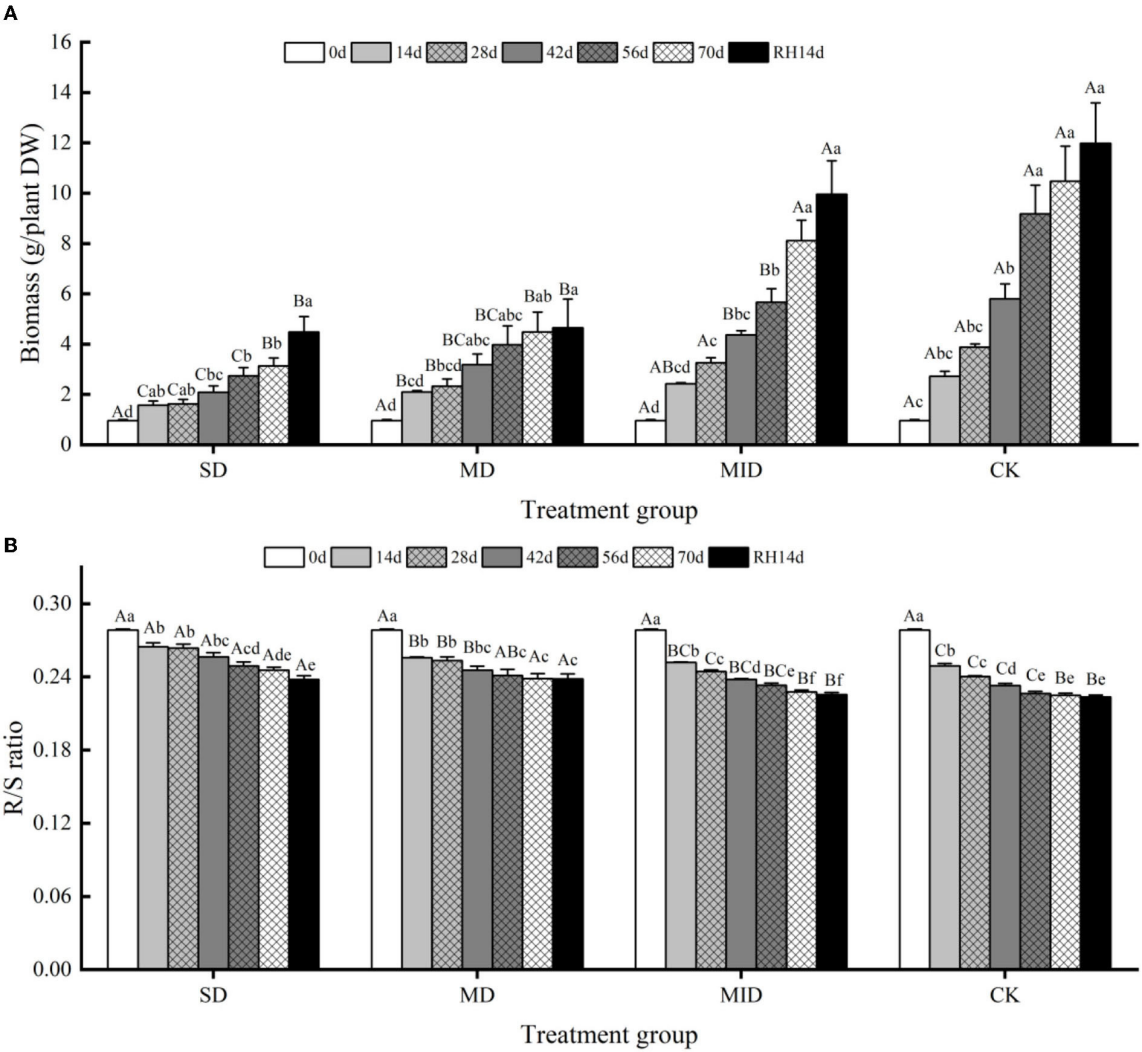
The biomass and R/S ratio during the drought and rehydration for *A. selengensis*. 0 day, 14 days, 28 days, 42 days, 56 days, 70 days, and RH, 14 days, respectively, represent 0th, 14th, 28th, 42nd, 56th, 70th under drought and 14th day of rehydration. The severe drought group (SD), the moderate drought group (MD), the mild drought group (MID), the control group (CK). **(A)** Total biomass of *A. selengensis*. **(B)** The R/S ratio of *A. selengensis*. Different capital letters stand for significant differences between different drought groups (*p* < 0.05), and different lowercase letters stand for significant differences between different experimental times (*p* < 0.05).

The effects of drought and rehydration on the biomass of *A. selengensis* are shown in Figure 5A, and the biomass showed an increasing trend. The effect of drought and rehydration on biomass accumulation varied among the four groups, and the degree of impact varied with the changes in drought intensity and duration. On day 28 of the experiment, the biomass of SD, MD, MID, and CK was 2.09, 3.18, 3.26, and 3.88 g/plant, respectively. At this time, the biomass of SD was significantly different from the other groups (*p* < 0.05, *p* < 0.001, *p* < 0.001), and there was no significant difference between the MID and CK (*p* > 0.05). This shows that the growth of plants subjected to severe drought was inhibited during the early growth period, resulting in slow biomass accumulation. On Day 70 of drought, the biomass of SD, MD, MID, and CK was 3.13, 4.48, 8.11, and 10.48 g/plant, respectively, and the biomass of four experimental groups was differentiated. The biomass accumulation of SD was at an equal level with MD, and the differences between MID and CK were also slight. It indicates that the biomass accumulation of MD was also strongly affected by the lengthening of drought time in the later stage. The biomass of SD and MD was significantly different from the CK (*p* < 0.001, *p* < 0.01), while the biomass of MID and CK was not significantly different (*p* > 0.05), suggesting that mild drought had little effect on the growth of *A. selengensis*.

After rehydration, the biomass of *A. selengensis* continued to increase, but its responses to rehydration differed in different groups. On day 14 of rehydration, the biomass of SD, MD, MID, and CK was 4.48, 4.65, 9.95, and 11.98 g/plant, respectively, and their biomass was 37.4, 38.81, and 83.1% of CK, respectively. After rehydration, the biomass of SD and MD was highly significantly different from the CK (*p* < 0.01), and the difference between the MID and CK was not significant (*p* > 0.05). It indicates that the biomass of *A. selengensis* can largely return to the control level under mild drought, but it is difficult to recover to natural with an increasing drought degree.

The response of *A. selengensis* R/S to drought and rehydration is shown in Figure 5B; the R/S showed a decreasing trend with the plants growing. On days 14, 28, and 42 of the experiment, the R/S of SD was significantly different from the other three groups (*p* < 0.001), and the differences between the SD and MD at the later stages of drought were not significant (*p* > 0.05). The difference in R/S between MID and CK was not significant, and their trends were similar. On day 70, the R/S of the four groups was 0.2455, 0.2385, 0.2278, and 0.2249, respectively, and the R/S of the SD and MD was significantly different from the MID and CK (*p* < 0.05, *p* < 0.01). It indicates that, at higher drought levels, the biomass allocation strategy of *A. selengensis* changes, thus increasing the proportion of root biomass accumulation. Therefore, the higher drought level, the higher R/S of *A. selengensis*.

The R/S continued to decrease after rehydration. On Day 14 of rehydration, the R/S of SD, MD, MID, and CK was 0.2380, 0.2382, 0.2255, and 0.2235, respectively. The R/S of SD and MD was highly significantly different from the CK (*p* < 0.001), and the MID was not significantly different from the CK (*p* > 0.05). This indicates that the R/S of *A. selengensis* under mild drought could recover to the control level after rehydration, but the R/S under severe drought and moderate drought was significantly higher than the control group and difficult to recover.

### Effects of drought and rehydration on relative water content and photosynthetic pigments of *A. selengensis*

#### Effects of drought and rehydration on relative water content of *A. selengensis*

**Figure 6:**
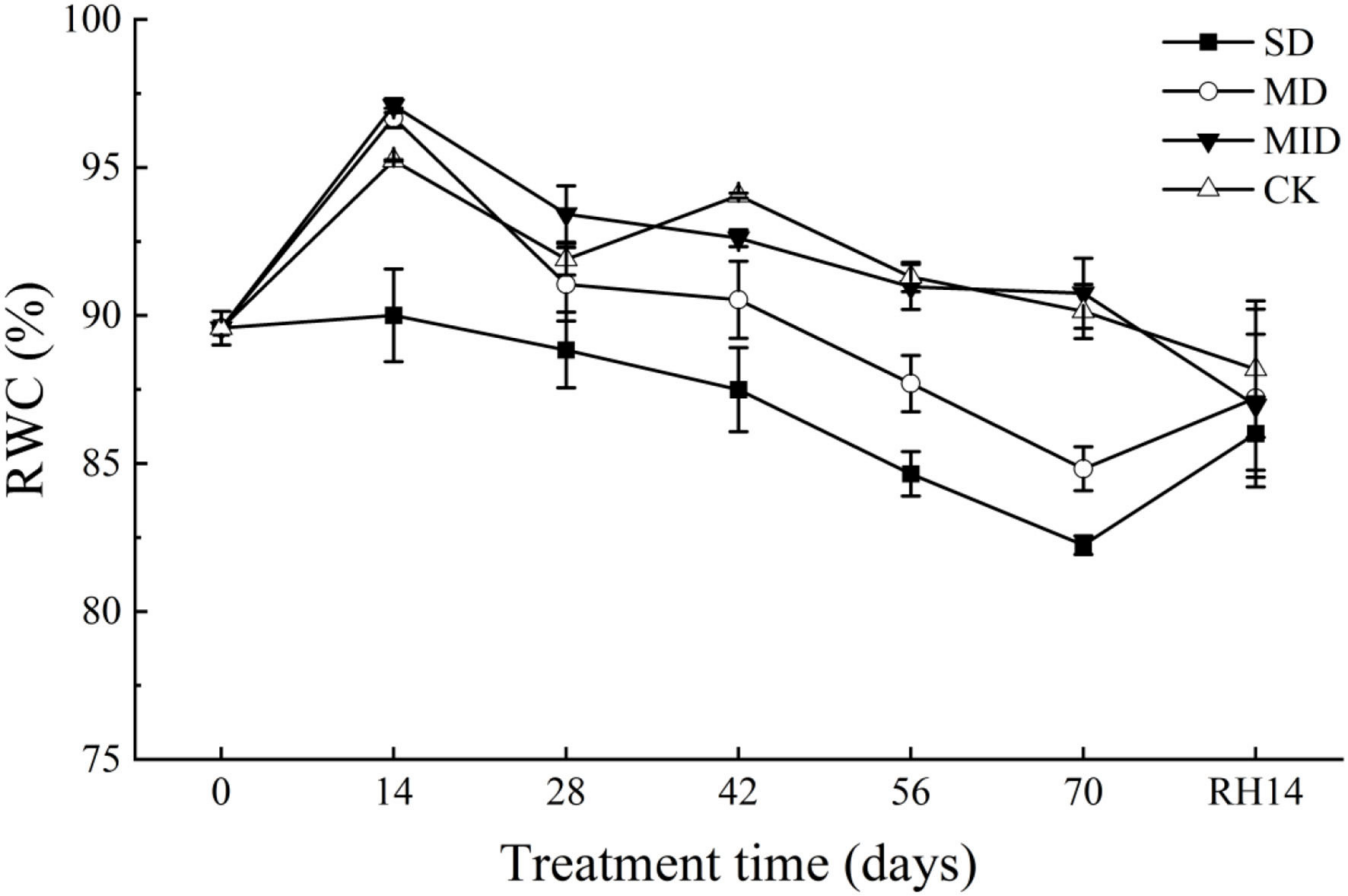
RWC during the drought and rehydration for *A. selengensis*. Fourteenth day of rehydration (RH, 14 days), the severe drought group (SD), the moderate drought group (MD), the mild drought group (MID), and the control group (CK).

The effects of drought and rehydration on RWC of *A. selengensis* leaves are shown in Figure 6. The RWC showed an increasing trend from Days 0–14 of the experiment and reached a peak on Day 14. During this time, the RWC of the SD, MD, MID, and CK was 90.03, 96.67, 97.09, and 95.24%, respectively. We found that the RWC of leaves in the SD was much lower than in other groups, with significant differences (*p* < 0.05). With the prolonged drought time, the RWC of all groups showed a decreasing trend and reached the lowest value on Day 70. As shown in Table 2, the RWC of leaves in *A. selengensis* showed a two-polar state under drought, the RWC of SD and MD was low, and the difference between the two groups with CK was significant (*p* < 0.05), while the RWC of the MID and CK was relatively high, and there was no significant difference betweenbr them (*p* > 0.05).

**Table 2:** RWC and photosynthetic pigment content of *A. selengensis* leaves after drought (Day 70) and rehydration (RH, 14 d).

**Time**	**Indicators**	**SD**	**MD**	**MID**	**CK**
Day 70	RWC	82.24 ± 0.30%b	84.82 ± 0.74%b	90.75 ± 1.18%a	90.14 ± 0.92%a
	Chl-a	5.19 ± 0.00c	5.54 ± 0.00b	4.65 ± 0.01d	8.83 ± 0.02a
	Chl-b	3.05 ± 0.01b	2.70 ± 0.00c	1.77 ± 0.00d	3.95 ± 0.02a
	Chl	8.25 ± 0.01b	8.24 ± 0.24b	6.42 ± 0.25c	12.79 ± 0.03a
	Car	0.25 ± 0.00c	0.34 ± 0.00b	0.26 ± 0.00c	0.57 ± 0.00a
RH 14d	RWC	86.01 ± 1.23%a	87.21 ± 3.00%a	86.95 ± 2.42%a	88.19 ± 2.30%a
	Chl-a	5.57 ± 0.02b	7.54 ± 0.00a	2.26 ± 0.00d	3.59 ± 0.01c
	Chl-b	3.45 ± 0.03a	3.44 ± 0.03a	0.97 ± 0.01c	3.32 ± 0.01b
	Chl	9.03 ± 0.01b	10.99 ± 0.03a	3.23 ± 0.01d	6.91 ± 0.02c
	Car	0.32 ± 0.02d	0.92 ± 0.01a	0.44 ± 0.00c	0.56 ± 0.00b

SD, severe drought; MD, moderate drought; MID, mild drought; CK, control group; RWC, relative water content, %; Chl-a, chlorophyll a, mg/gDW; Chl-b, chlorophyll b, mg/gDW; Chl, chlorophyll, mg/gDW; Car, carotenoid, mg/gDW. Different letters stand for significant differences between different drought groups (p <0.05).

After 14 days of rehydration, the RWC of leaves in the SD and MD began to increase, while the MID and CK continued to decrease. At this time, the RWC of all groups was very closed, and, as shown in Table 2, the differences between the groups were not significant (*p* > 0.05) after rehydration. The above results indicate that the RWC of *A. selengensis* is affected by drought, resulting in lower RWC than CK. After rehydration, the RWC of leaves in the severe drought, moderate drought, and mild drought all returned to normal levels, showing a better recovery ability.

#### Effects of drought and rehydration on the photosynthetic pigment of *A. selengensis*

**Figure 7:**
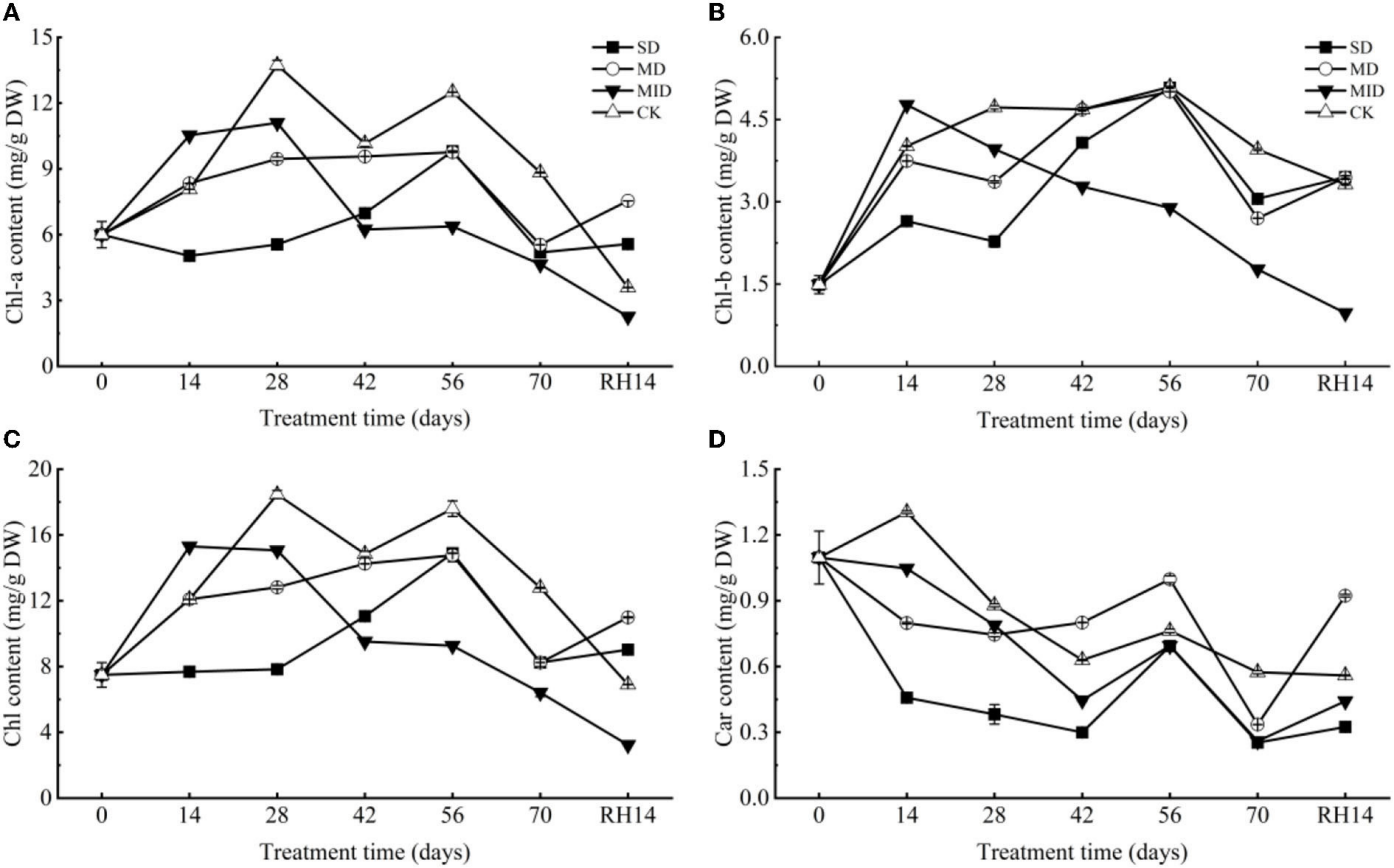
The photosynthetic pigment during the drought and rehydration for *A. selengensis*. Fourteenth day of rehydration (RH, 14 days), chlorophyll a (Chl-a), chlorophyll b (Chl-b), total chlorophyll (Chl), carotenoids (Car). The severe drought group (SD), the moderate drought group (MD), the mild drought group (MID), the control group (CK). **(A)** Chl-a of *A. selengensis*. **(B)** Chl-b of *A. selengensis*. **(C)** Chl of *A. selengensis*. **(D)** Car of *A. selengensis*.

As shown in Figure 7A, Chl-a content showed a trend of, firstly, increasing and then decreasing. However, the time of their peak values differed, with the Chl-a in MID and CK, showing a peak value of 11.10 and 13.74 mg/g on Day 28, respectively, the SD and MD showing a peak value of 9.81 and 9.76 mg/g on Day 56, respectively. The Chl-a of SD was decreased and significantly lower than that of CK (*p* < 0.001) during 0–14 days, whereas MD and MID were significantly higher than CK (*p* < 0.001). The Chl-a content of SD, MD, and MID was significantly lower than that of CK during 14–70 days (*p* < 0.001), while SD and MD were significantly higher than MID (*p* < 0.05). At the end of drought treatment, the Chl-a content of SD, MD, and MID was significantly lower than that of CK (*p* < 0.001). After 14 days of rehydration, the Chl-a content in MID and CK maintained a decreasing trend, while that in SD and MD increased. At this time, the Chl-a content of SD and MD was significantly higher than that of CK (*p* < 0.001), and MID was significantly lower than CK (*p* < 0.001).

The changes of Chl-b in *A. selengensis* are shown in Figure 7B. Under drought, Chl-b content showed a trend of increasing and then decreasing, with the MID reaching the maximum value of 4.77 mg/g on Day 14. The peak of SD, MD, and CK occurred on Day 56, with Chl-b content of 5.08, 5.01, and 5.10 mg/g, respectively. The Chl-b content of MID was significantly higher than CK on Days 0–14 (*p* < 0.001), while SD and MD were significantly lower than CK (*p* < 0.001). Chl-b content of MID decreased continuously and was significantly lower than CK after 14 days of the experiment (*p* < 0.001), while SD and MD showed an increasing trend on Days 14–56, and MD and SD on Days 42 and 56, respectively, reached the same level as CK (*p* > 0.05). But as the drought time was prolonged, the Chl-b content of both SD and MD decreased and was significantly lower than CK (*p* < 0.001) on Days 56–70. After rehydration, the Chl-b content of MID and CK continued to decrease, while that of SD and MD increased. At this time, Chl-b of MID was significantly lower than that of CK (*p* < 0.001), but SD and MD were significantly higher than that of CK (*p* < 0.01).

The change of Chl of *A. selengensis* is shown in Figure 7C. The trend of Chl during the drought was similar to Chl-a and Chl-b, which both firstly increased and then decreased. The content of Chl in the MID reached a peak of 15.30 mg/g on Day 14, the CK reached a peak of 18.46 mg/g on Day 28, and both SD and MD reached respectively their highest values of 14.89 mg/g and 14.77 mg/g on Day 56. On Day 14 of the experiment, Chl in SD was significantly lower than that in CK (*p* < 0.001), while those in MD and MID were higher than that in CK. But at 14–70 days, Chl in all drought treatments was significantly lower than CK (*p* < 0.01). The Chl content of SD and MD was very close (*p* > 0.05) on Days 56–70, and both were significantly higher than that of MID (*p* < 0.001). After rehydration, the Chl content in the SD and MD exceeded the CK, while the MID was lower (*p* < 0.001).

As presented in Figure 7D, the content of Car exhibited an overall decreasing trend. Car content in all drought groups was significantly lower (*p* < 0.01) than CK during Days 0–28 of the experiment, and its content decreased with increasing drought intensity. On Days 42–56, the Car content in the SD, MD, and MID increased and then decreased, and the Car content of MD was higher than that of CK at this time (*p* < 0.001). At the end of the drought treatment, the Car content of SD, MD, and MID was significantly lower than that of CK (*p* < 0.001). After rehydration, the Car content of the CK was generally stable, while the drought group started to increase, especially the Car content of MD already surpassed the CK (*p* < 0.001).

### Effects of drought and rehydration on osmolytic substances in *A. selengensis*

#### The effect of drought and rehydration on soluble protein

**Figure 8:**
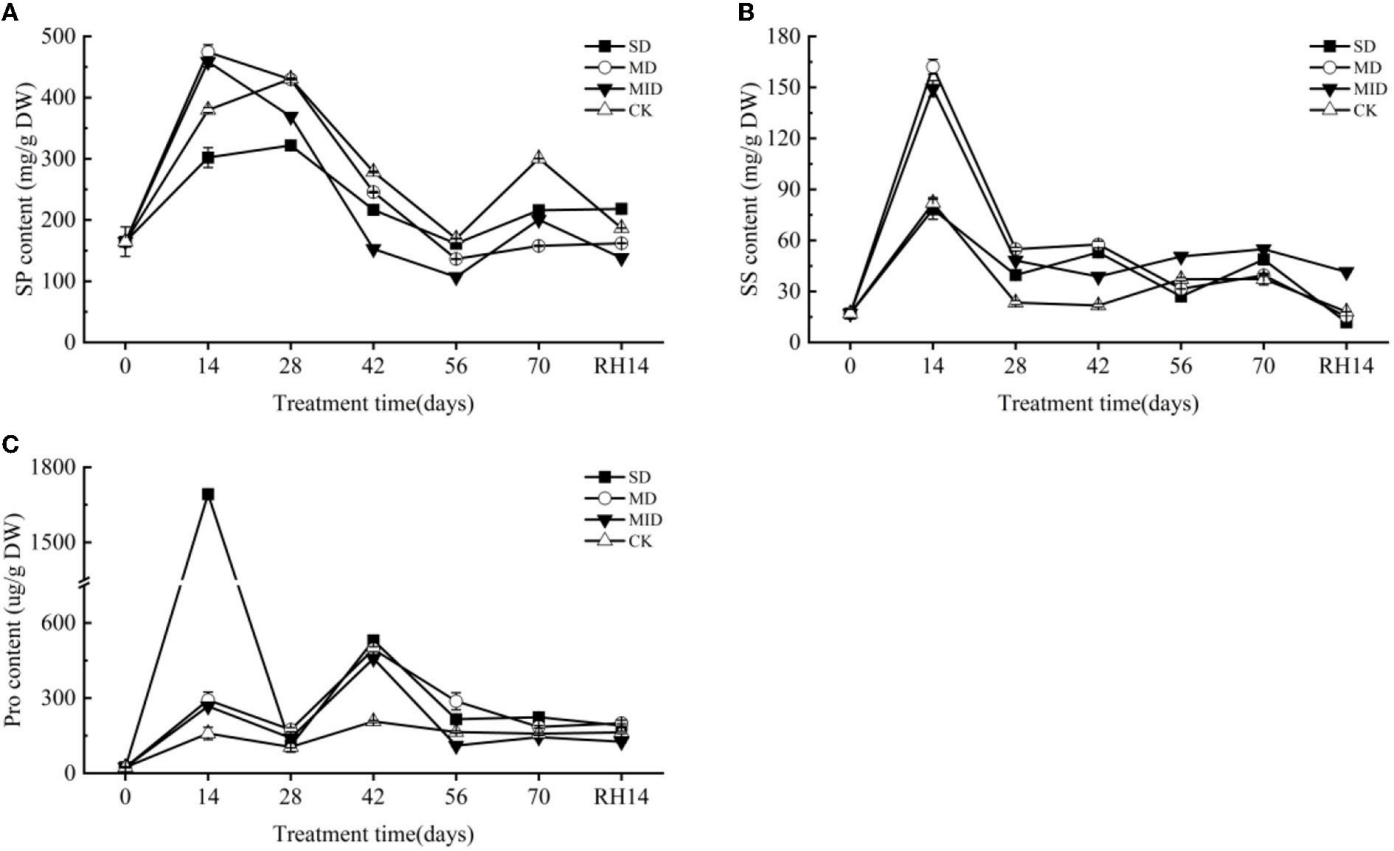
Osmolytic substances during the drought and rehydration for *A. selengensis*. Fourteenth day of rehydration (RH, 14 days), soluble protein (SP), soluble sugar (SS), proline (Pro). The severe drought group (SD), the moderate drought group (MD), the mild drought group (MID), the control group (CK). **(A)** SP of *A. selengensis*. **(B)** SS of *A. selengensis*. **(C)** Pro of *A. selengensis*.

The effects of drought and rehydration on the SP content of *A. selengensis* are shown in Figure 8A. The SP content of each treatment group increased sharply at the early stage of drought. The SP content of MD and MID reached a maximum of 474.38 and 458.58 mg/g at 14 days and was significantly higher than that of CK (*p* < 0.01). On Day 28, the SP content of SD peaked at 321.87 mg/g, and the SP content in the SD and MID was significantly lower than that of the CK (*p* < 0.001). On Day 56, the SP content of drought groups reached the lowest value of 161.44, 136.34, and 106.95 mg/g, respectively, and the SP content of CK was significantly higher than other groups (*p* < 0.001). As shown in Table 3, the SP contents of the SD, MD, MID, and CK were 215.94, 157.71, 200.52, and 300.52 on Day 70, respectively, with highly significant differences between the groups (*p* < 0.001). It is worth mentioning that the SP content of MD and MID was higher than CK at Days 0–14, but they are also gradually lower than CK with time.

**Table 3:** Osmotic regulator substance content in leaves of *A. selengensis* after drought (Day 70) and rehydration (RH, 14 d).

**Time**	**Indicators**	**SD**	**MD**	**MID**	**CK**
Day 70	SP	215.94 ± 1.08b	157.71 ± 0.97d	200.52 ± 1.73c	300.52 ± 0.34a
	SS	48.78 ± 2.64a	39.47 ± 0.52b	54.89 ± 1.00a	37.28 ± 3.47b
	Pro	223.63 ± 4.24a	185.21 ± 4.74b	144.04 ± 6.83c	157.88 ± 4.47c
RH 14d	SP	218.29 ± 0.83a	161.97 ± 0.64c	138.14 ± 0.10d	186.87 ± 0.18b
	SS	11.79 ± 0.04d	15.22 ± 0.00c	41.49 ± 0.10a	18.04 ± 0.05b
	Pro	189.95 ± 7.81ab	199.66 ± 12.47a	125.99 ± 3.68c	162.94 ± 5.01b

SD, severe drought; MD, moderate drought; MID, mild drought; CK, control group; SP, soluble protein, mg/g DW; SS, soluble sugar, mg/g DW; Pro, proline, ug/g DW. Different letters stand for significant differences between different drought groups (p < 0.05).

After rehydration, the SP content in MID and CK appeared a decreasing trend, but that of SD and MD was not decreased. As shown in Table 3, the content of SP was 218.29, 161.97, 138.14, and 186.87 mg/g in the SD, MD, MID, and CK after rehydration, respectively. The SP content of SD was extremely significantly higher than the CK (*p* < 0.001), but the SP content of MD and MID was significantly lower than the CK (*p* < 0.001). It indicates that the SP content under MD was fully recovered.

#### The effect of drought and rehydration on soluble sugar

The changes in SS content of *A. selengensis* are shown in Figure 8B. On Day 14, the SS content began to rise and reached its peak, and the SS content of each group was 78.30, 162.14, 149.24, and 82.01 mg/g, respectively. The SS content in the MD and MID was significantly higher than in the CK (*p* < 0.001), and there was no significant difference between the SD and CK (*p* > 0.05). On Days 14–56, SS showed a decreasing trend in SD and MD, and even significantly lower than that of CK on Day 56 (*p* < 0.001). As shown in Table 3, the SS content at Day 70 was 48.78, 39.47, 54.89, and 37.28 mg/g in the SD, MD, MID, and CK, respectively, and the SS content of SD and MID was significantly higher than that of the CK (*p* < 0.01). It can be found the SS content under drought was generally higher than the normal level.

After rehydration, the content of SS in all groups decreased, and they were 1,179, 15.22, 41.49, and 18.04 mg/g, respectively. As shown in Table 3, the SS content of SD and MD was slightly lower than the CK, and the MID was much higher than CK (*p* < 0.001). The SS content of the MID did not return to the control level, and the SS content of SD and MD was at a relatively low level and recovered to normal.

#### The effect of drought and rehydration on proline

The effect of drought and rehydration on the Pro content of *A. selengensis* is shown in Figure 8C. The content of Pro generally showed a trend of increasing first and then decreasing. The peak of Pro content in the SD appeared on Day 14. The Pro content at this time was 1,692.75 ug/g, which was several times higher than other treatment groups; there was a very significant difference (*p* < 0.001). The peak value of MD, MID, and CK appeared on Day 42; the Pro content of the three groups was 494.86, 458.01, and 206.76 ug/g, respectively. The Pro content of CK is much lower than drought groups on Days 0–42, and there was a significant difference at 14 and 42 days (*p* < 0.001). As shown in Table 3, the order of Pro content in each group was SD > MD > CK > MID, and the Pro content of SD and MD was significantly higher than that of CK (*p* < 0.01) on Day 70. The above results indicate that SD and MD have caused stress to *A. selengensis*, which produces more proline to cope with the drought stress.

After rehydration, the Pro content of the SD, MD, MID, and CK was 189.95, 199.66, 125.99, and 162.94 ug/g, and their orders at this time were MD > SD > CK > MID (Table 3). We can find that the Pro content of SD and MD is significantly higher than that of CK (*p* < 0.05), and that of MID was significantly lower than CK (*p* < 0.05). It shows that the Pro content of SD and MD groups may take a longer time to recover to the control level.

## Discussion

Drought stress decreased the plant growth rate as well as biomass accumulation in different parts of the plant (Husen et al., 2014), leading to prolonged growth time (Verbraeken et al., 2021). In this study, the plant height, basal diameter, biomass, and R/S ratio of *A. selengensis* were significantly affected by the drought level and duration (Table 1). The plant height, basal diameter, and biomass under SD and MD were severely restricted (Figures 3, 4, 5A), while the R/S ratio increased significantly (Figure 5B). However, these traits were less affected under MID conditions. The results indicate that SD and MD caused stress to the growth of *A. selengensis*, and the plant adjusted its biomass allocation strategy to adapt to the drought conditions. Although the plant root system directly perceives soil water deficit, previous studies have suggested that drought stress inhibits plant aboveground biomass accumulation more than that of the root system, thus leading to an increase in the R/S ratio (Pace and Benincasa, 2010). The studies on rice showed a significant increase in the R/S ratio under drought stress, especially severe drought, compared to well-watered rice (Xu et al., 2015). This study concluded that the increase in R/S was largely attributed to a decrease in aboveground biomass rather than increased root biomass. Studies on fennel suggested that water deficit may increase root growth and rooting depth, thus maintaining or increasing the biomass allocated to the roots in response to water limitation (Askari and Ehsanzadeh, 2015). Reallocation of plant biomass is considered to be a favorable adaptation mechanism for plants to reduce the evapotranspiration area of the leaf canopy while increasing the ability to absorb water from the soil (Mahajan and Tuteja, 2005). In our study, the order of the R/S ratio under different water conditions was SD > MD > MID > CK; the R/S was positively correlated with the degree of drought within a limited range. After rehydration, the plant height, basal diameter, biomass, and R/S of *A. selengensis* in the MID could be restored to the control level, but, under that of the SD and MD, could not be restored. The level and duration of drought treatment had significant effects on morphological indicators of *A. selengensis* (Table 1), and rehydration is more effective in the early stages of plant growth than in the middle and late stages (Zhang et al., 2019). Thus, excessive stress and duration may be the reason for failure to recover. To sum up, the *A. selengensis* could survive the drought period by increasing the proportion of root biomass, which showed morphological adaptability to drought resistance. However, only the morphological changes induced by mild drought could be recovered after rehydration.

Drought stress is one of the major abiotic factors limiting plant growth, which can have important effects on plant physiology and biochemistry. Photosynthesis is extremely sensitive to water stress (Chaitanya et al., 2003), and drought-induced reduction in chlorophyll content is very common in different plants (Sun et al., 2013). Some studies have revealed that drought induces a strong reduction of chlorophyll content in maize leaves, a significant loss of photosynthetic reaction centers; and carotenoid content also declines along with chlorophyll, because carotenoids are mainly associated with photosynthetic reaction centers. However, after rehydration, photosynthetic pigments in the leaves are restored in time (Sun et al., 2018). The chlorophyll content of the herbaceous plant purslane (*Portulaca oleracea* L.) gradually decreases under drought and is also restored after rehydration (Jin et al., 2015). Chlorophyll loss is a negative consequence of plant stress on plants, but it is also considered an adaptive feature because it reduces light harvesting as well as the possibility of further damage to the photosynthetic machinery caused by activated oxygen radicals in case of excess excitation energy (Munne-Bosch and Alegre, 2000; Kranner et al., 2002). On the contrary, there are also studies showing that the chlorophyll content of plants with higher drought tolerance increases with increasing drought intensity (An et al., 2011; Bortolheiro and Silva, 2017; Chen J. et al., 2021). In our study, drought levels and duration had significant effects on photosynthetic pigments (Table 1). Drought did reduce chlorophyll content of SD and MD, but chlorophyll content in SD and MD exceeded that of MID at the late stage of drought (Figure 7). This indicates that *A. selengensis* in the SD and MD groups started to adapt to drought with time, such as by increasing the R/S ratio to reduce evaporation. Previous drought experiments on crops such as rice indicated that drought-sensitive rice genotypes lost up to three times more carotenoids compared to drought-tolerant genotypes of rice (Chutipaijit et al., 2012). Carotenoids are the most important physiological markers of drought tolerance for the evaluation of alfalfa, in priority to soluble sugars and RWC (Maghsoodi and Razmjoo, 2015). The carotenoid content of *A. selengensis* fluctuated, but, in general, it was lower in the drought groups. Carotenoids have antioxidant effects, and the reduction in their content may be due to their oxidation by singlet oxygen (Gori et al., 2021), an adaptation of *A. selengensis* to reduce oxidative damage. After rehydration, the chlorophyll content of *A. selengensis* was higher in the SD and MD than in the CK. The carotenoid content was increased in the SD, MID, and MD, and that of MD was significantly higher than CK (Table 2). This indicates that the photosynthetic pigments of plants could be fully or partially recovered after the removal of stress and even higher than the control level because of the compensatory effect, which is consistent with the chlorophyll content of *Haberlea rhodopensis* under drought in response to rehydration (Georgieva et al., 2012). In summary, the loss of photosynthetic pigments in *A. selengensis* under drought was significant, but the photosynthetic pigments under SD and MD could be restored to normal levels after rehydration.

The leaf RWC of water-deficient plants tends to be below 80% (Batool et al., 2020); the RWC of safflower under water deficit conditions can decrease to 45.9% on average (Bortolheiro and Silva, 2017). During the drought, leaf RWC under SD and MD was significantly lower than that under MID and CK, with an overall decreasing trend of RWC in all groups, but still maintaining a high RWC (Figure 6). Some studies on wheat have shown that drought-tolerant varieties of wheat maintain 90% of RWC after drought (Zhan et al., 2015; Yadav et al., 2019). The RWC of 17 cultivars of potatoes ranged from 64.4 to 86.7% under drought, while the highest RWC was 92%, which was not significantly affected by drought (Soltys-Kalina et al., 2016). *A. selengensis* can also maintain a high RWC for a longer period, indicating its high drought tolerance. Drought-induced reduction in stem elongation and the increased R/S ratio contribute to the maintenance of RWC (Omae et al., 2015); thus, the changes in plant height and the R//S ratio may contribute to water retention in *A. selengensis* (Figures 3, 5B). After rehydration, the RWC of *A. selengensis* with a higher degree of drought was restored and exceeded the CK and MID, reflecting the compensatory effect of rehydration on plants (Table 2). Previous studies have shown that leaf RWC of *Caragana korshinskii Kom* declined sharply under extreme drought, eventually leading to leaf abscission (Xu et al., 2012). Moreover, plants subjected to drought stress generally had lower RWC than the control and recovered somewhat after rehydration (Upadhyaya and Panda, 2004; Benetti Mantoan et al., 2016), which is consistent with our findings for *A. selengensis*. These results indicate that RWC of *A. selengensis* can not only be maintained at a high level under drought conditions but also recover quickly after rehydration under SD and MD.

*A. selengensis* maintains a high RWC not only by morphological adaptation but also by osmotic adjustment (Serraj and Sinclair, 2002). SS is highly sensitive to environmental stress, and environmental stress affects the supply of carbohydrates from source organs to sink organs (Rosa et al., 2009). Pro is considered to have a positive effect on enzymes and cell membrane integrity and has an adaptive function in regulating osmoregulation in plants grown under stress conditions (Ashraf and Foolad, 2007). In this study, SS and Pro accumulation increased sharply in all drought groups compared to in CK (Figures 8B,C), and the contents of SS and SP in the drought groups were significantly higher than those in CK (Table 3). Proline and sugar accumulation in strawberry leaves subjected to drought stress was higher than in the control (Sun et al., 2015), which is consistent with our results. However, studies on rice seedlings concluded that drought stress leads to a significant reduction in the accumulation of soluble sugars in the whole plant (Xu et al., 2015; Dien et al., 2019). This is because SS, including monosaccharides and oligosaccharides, is the main product of photosynthesis (Bodelón et al., 2010), but photosynthesis in plants is inhibited during drought. The MID and MD exhibited higher SS accumulation than the SD, indicating that the soluble sugar synthesis process was restricted and disrupted earlier in *A. selengensis* under severe drought. In addition, *A. selengensis* under SD and MD had an increased R/S ratio (Figure 5B) and reduced aboveground parts, which further weakened photosynthesis and led to the limitation of sugar synthesis. After rehydration, the Pro content of MID and the SS content of SD and MD were lower than that of the control level. Similar to our results, Pro content in wheat under drought also decreased substantially after rehydration (Maevskaya and Nikolaeva, 2013). This is because the Pro in the plant is converted to glutamate by proline dehydrogenase (PDH) and P5C dehydrogenase (P5CDH) (Verbruggen and Hermans, 2008). The Pro content of SD and MD is also decreasing, but it may take longer to reach the control level.

Different from SS and Pro, we found that the SP content of SD was lower than that of CK at the early stage of drought. With the prolongation of drought, the SP content of MD and MID was also lower than that of CK (Figure 8A), and that of all drought groups was significantly lower than CK at the end of the drought (Table 3). It was not consistent with most previous studies (Guo et al., 2018; Wang et al., 2021). The most plausible explanation is that, when drought stress becomes more severe, plants maintain their vital metabolic activities through protein degradation (Reddy et al., 2004), leading to a decrease in protein content, which is also similar to the results of Dendrobium moniliforme (Wu et al., 2016). It is also suggested that drought inhibits protein synthesis, resulting in lower SP content in plants (Wang et al., 2019). After rehydration, the SP content of the MD and MID is lower than the CK, implying that rehydration even produces a compensation or overcompensation effect (Wang et al., 2021). In summary, *A. selengensis* can respond to the threat of drought by regulating osmotic substances, and this regulation includes increasing SS and Pro accumulation to maintain osmotic pressure and RWC and depleting SS and SP to sustain vital activities.

## Conclusion

In this study, the effects of wetland water deficit and rehydration processes on the morpho-physiology of *A. selengensis* were simulated. After analyzing the results, we believe that Hypotheses (1) and (2) raised previously have been partially or fully verified. Drought inhibited the increase in plant height, basal diameter, and biomass. In addition, drought reduces Chl and Car content. The inhibition became more significant as the stress became more severe and prolonged. However, *A. selengensis* was able to maintain high levels of RWC by increasing the R/S ratio, SS, and Pro. After rehydration, RWC, Chl, Car, SP, SS, and Pro were fully or partially recovered, and the content of photosynthetic pigments and osmotic substances was even partially compensated. In conclusion, *A. selengensis* is highly resistant to drought, adopts multiple adaptive strategies to cope with drought, and activates many physiological mechanisms to achieve more effective recovery during rehydration. Therefore, we predict that *A. selengensis* may benefit from possible future aridification of wetlands and expand population distribution.

## Data availability statement

The original contributions presented in the study are included in the article/supplementary material, further inquiries can be directed to the corresponding author.

## Author contributions

YCa and HH designed the experiment. HH, YCh, and JQ conducted the experiment and analyzed the data. HH composed the manuscript. YCa, KX, and RL revised the manuscript. All authors read and approved the final manuscript.

## Funding

This work was supported by grant from the National Natural Science Foundation of China (42061021).

## Conflict of interest

The authors declare that the research was conducted in the absence of any commercial or financial relationships that could be construed as a potential conflict of interest.

## Publisher's note

All claims expressed in this article are solely those of the authors and do not necessarily represent those of their affiliated organizations, or those of the publisher, the editors and the reviewers. Any product that may be evaluated in this article, or claim that may be made by its manufacturer, is not guaranteed or endorsed by the publisher.
